# Variation of sperm quality and circular RNA content in men exposed to environmental contamination with heavy metals in ‘Land of Fires’, Italy

**DOI:** 10.1093/humrep/deae109

**Published:** 2024-06-17

**Authors:** Vincenza Grazia Mele, Teresa Chioccarelli, Nadia Diano, Donato Cappetta, Bruno Ferraro, Marialucia Telesca, Martina Moggio, Veronica Porreca, Antonella De Angelis, Liberato Berrino, Silvia Fasano, Gilda Cobellis, Rosanna Chianese, Francesco Manfrevola

**Affiliations:** Department of Experimental Medicine, University of Campania L. Vanvitelli, Naples, Italy; Department of Experimental Medicine, University of Campania L. Vanvitelli, Naples, Italy; Department of Experimental Medicine, University of Campania L. Vanvitelli, Naples, Italy; Department of Experimental Medicine, University of Salento, Lecce, Italy; UOSD of Reproductive Pathophysiology, Marcianise Hospital, Caserta, Italy; Department of Experimental Medicine, University of Campania L. Vanvitelli, Naples, Italy; Department of Experimental Medicine, University of Campania L. Vanvitelli, Naples, Italy; Department of Experimental Medicine, University of Campania L. Vanvitelli, Naples, Italy; Department of Experimental Medicine, University of Campania L. Vanvitelli, Naples, Italy; Department of Experimental Medicine, University of Campania L. Vanvitelli, Naples, Italy; Department of Experimental Medicine, University of Campania L. Vanvitelli, Naples, Italy; Department of Experimental Medicine, University of Campania L. Vanvitelli, Naples, Italy; Department of Experimental Medicine, University of Campania L. Vanvitelli, Naples, Italy; Department of Experimental Medicine, University of Campania L. Vanvitelli, Naples, Italy

**Keywords:** circular RNA, environmental contaminants, fused in sarcoma protein, heavy metals, Land of Fires, lead, male infertility, spermatozoa

## Abstract

**STUDY QUESTION:**

Can illegal discharge of toxic waste into the environment induce a new condition of morpho-epigenetic pathozoospermia in normozoospermic young men?

**SUMMARY ANSWER:**

Toxic environmental contaminants promote the onset of a new pathozoospermic condition in young normozoospermic men, consisting of morpho-functional defects and a sperm increase of low-quality circular RNA (circRNA) cargo, tightly linked to contaminant bioaccumulation in seminal plasma.

**WHAT IS KNOWN ALREADY:**

Epidemiological findings have reported several reproductive anomalies depending on exposure to contaminants discharged into the environment, such as germ cell apoptosis, steroidogenesis defects, oxidative stress induction, blood–testis barrier dysfunctions, and poor sperm quality onset. In this scenario, a vast geographical area located in Campania, Italy, called the ‘Land of Fires’, has been associated with an excessive illegal discharge of toxic waste into the environment, negatively impacting human health, including male reproductive functions.

**STUDY DESIGN, SIZE, DURATION:**

Semen samples were obtained from healthy normozoospermic men divided into two experimental groups, consisting of men living in the ‘Land of Fires’ (LF; n = 80) or not (CTRL; n = 80), with age ranging from 25 to 40 years. The study was carried out following World Health Organization guidelines.

**PARTICIPANTS/MATERIALS, SETTING, METHODS:**

Quality parameters of semen from CTRL- and LF-normozoospermic men were evaluated by computer-assisted semen analysis; high-quality spermatozoa from CTRL and LF groups (n = 80 for each experimental group) were obtained using a 80–40% discontinuous centrifugation gradient. Seminal plasma was collected following centrifugation and used for the dosage of chemical elements, dioxins and steroid hormones by liquid chromatography with tandem mass spectrometry. Sperm morpho-functional investigations (cellular morphology, acrosome maturation, IZUMO1 fertility marker analysis, plasma membrane lipid state, oxidative stress) were assessed on the purified high-quality spermatozoa fraction by immunochemistry/immunofluorescence and western blot analyses. Sperm circRNA cargo was evaluated by quantitative RT-PCR, and the physical interaction among circRNAs and fused in sarcoma (FUS) protein was detected using an RNA-binding protein immunoprecipitation assay. Protein immunoprecipitation experiments were carried out to demonstrate FUS/p-300 protein interaction in sperm cells. Lastly, *in vitro* lead (Pb) treatment of high-quality spermatozoa collected from normozoospermic controls was used to investigate a correlation between Pb accumulation and onset of the morpho-epigenetic pathozoospermic phenotype.

**MAIN RESULTS AND THE ROLE OF CHANCE:**

Several morphological defects were identified in LF-spermatozoa, including: a significant increase (*P* < 0.05 versus CTRL) in the percentage of spermatozoa characterized by structural defects in sperm head and tail; and a high percentage (*P* < 0.01) of peanut agglutinin and IZUMO1 null signal cells. In agreement with these data, abnormal steroid hormone levels in LF seminal plasma suggest a premature acrosome reaction onset in LF-spermatozoa. The abnormal immunofluorescence signals of plasma membrane cholesterol complexes/lipid rafts organization (Filipin III and Flotillin-1) and of oxidative stress markers [3-nitrotyrosine and 3-nitrotyrosine and 4-hydroxy-2-nonenal] observed in LF-spermatozoa and associated with a sperm motility reduction (*P* < 0.01), demonstrated an affected membrane fluidity, potentially impacting sperm motility. Bioaccumulation of heavy metals and dioxins occurring in LF seminal plasma and a direct correlation between Pb and deregulated circRNAs related to high- and low-sperm quality was also revealed. In molecular terms, we demonstrated that Pb bioaccumulation promoted FUS hyperacetylation via physical interaction with p-300 and, in turn, its shuttling from sperm head to tail, significantly enhancing (*P* < 0.01 versus CTRL) the endogenous backsplicing of sperm low-quality circRNAs in LF-spermatozoa.

**LIMITATIONS, REASONS FOR CAUTION:**

Participants were interviewed to better understand their area of origin, their eating habits as well as their lifestyles, however any information incorrectly communicated or voluntarily omitted that could potentially compromise experimental group determination cannot be excluded. A possible association between seminal Pb content and other heavy metals in modulating sperm quality should be explored further. Future investigations will be performed in order to identify potential synergistic or anti-synergistic effects of heavy metals on male reproduction.

**WIDER IMPLICATIONS OF THE FINDINGS:**

Our study provides new findings regarding the effects of environmental contaminants on male reproduction, highlighting how a sperm phenotype classified as normozoospermic may potentially not match with a healthy morpho-functional and epigenetic one. Overall, our results improve the knowledge to allow a proper assessment of sperm quality through circRNAs as biomarkers to select spermatozoa with high morpho-epigenetic quality to use for ART.

**STUDY FUNDING/COMPETING INTEREST(S):**

This study was supported by ‘Convenzione Azienda Sanitaria Locale (ASL) Caserta, Regione Campania’ (ASL CE Prot. N. 1217885/DIR. GE). The authors have no conflict of interest to declare.

**TRIAL REGISTRATION NUMBER:**

N/A.

## Introduction

A range of chemical compounds commonly used by modern industry is widely dispersed in the environment and appears, directly or indirectly, responsible for environmental contaminant (EC)-related disease onset, including male infertility (Cescon *et al.*, 2020; Selvaraju *et al.*, 2021; Wu *et al.*, 2023). In this context, a direct correlation between the exposure to highly heterogeneous ECs—[(i.e. heavy metals; polychlorinated biphenyls (PCBs); bisphenol A (BPA); polycyclic aromatic hydrocarbons; endocrine-disrupting (ED) chemicals)]—and the decline of male reproductive health has been well reported (Chianese *et al.*, 2018a,b; Street *et al.*, 2018; Cescon *et al.*, 2020; Chioccarelli *et al.*, 2020a; Selvaraju *et al.*, 2021). Indeed, several epidemiological and experimental studies have highlighted specific male reproductive defects depending on the type of contaminant and on the exposure window (Barakat *et al.*, 2019; Campos *et al.*, 2019; Selvaraju *et al.*, 2021). In particular, EC exposure impairs spermatogenesis and gamete health by inducing multiple anomalies, such as: deregulation of spermatogenic cycle timing; production of reactive oxygen species (ROS); Sertoli and germ cell apoptosis; germ cell abnormal chromosomal segregation and DNA fragmentation; steroidogenesis defects; disruption of blood-testis-barrier integrity and permeability; and alteration of sperm morphology and motility (Jeng, 2014; Easley *et al.*, 2015; Governini *et al.*, 2015; Qiu *et al.*, 2016; Aydin and Erkan, 2017; Jia *et al.*, 2017; Rodríguez *et al.*, 2017; Zhang *et al.*, 2018; Santoro *et al.*, 2019; Maske *et al.*, 2020; Chioccarelli *et al.*, 2021b).

Noteworthy is that EC exposure negatively affects the sperm epigenetic landscape leading to the production of spermatozoa with low epigenetic quality, which can impair offspring health *via* paternal transgenerational epigenetic inheritance mechanisms (Cescon *et al.*, 2020). In this regard, a large variety of EC adverse effects on sperm epigenome has emerged. In fact, sperm histone post-translational modifications, global DNA methylation as well as sperm RNA profile may be tightly influenced by a wide range of ECs (Cescon *et al.*, 2020). For example, hypomethylation of the H19-imprinted gene is directly promoted by dichlorodiphenoxydichloroethylene (Shi *et al.*, 2010, 2013), while carbendazim and chlorothalonil negatively affect both DNA and histone methylation (Liu *et al.*, 2019; Zhang *et al.*, 2019). BPA exposure promotes a global increase of genome-wide methylation in spermatocytes and deregulates H3K27Ac/H3K9Ac (histone acetylation) levels in spermatozoa (Yin *et al.*, 2016; Lombó *et al.*, 2019). In addition, the sperm protamine/histone ratio is affected, making spermatozoa more sensitive to DNA damage (Lettieri *et al.*, 2020a,b). Furthermore, cadmium, irradiation and ED chemicals induce an aberrant expression profile of long non-coding RNAs (lncRNAs) and mRNAs, as well as anomalies in sperm micro-RNA (miRNA) content (Filkowski *et al.*, 2010; Meunier *et al.*, 2012; Siddeek *et al.*, 2016; Gao *et al.*, 2017).

In this scenario, circular RNAs (circRNAs), a class of ncRNAs produced by backsplicing under the guidance of RNA-binding proteins (RBPs), such as fused in sarcoma (FUS) protein and quaking (QKI), are acquiring a prominent role in the molecular and morphological sperm quality setting. Our previous findings have, in fact, highlighted a differential circRNA expression profile in human spermatozoa depending on sperm quality evaluated in terms of structural integrity and motility (Chioccarelli *et al.*, 2019). Considering the peculiar circRNA localization in sperm head and tail subcellular fractions, circRNA transfer from spermatozoa to the oocyte during fertilization appears to be a molecular mechanism that is key for supporting embryo development. According to this hypothesis, we have previously demonstrated the increase of a specific sperm circRNA (circNAPEPLDiso1)—able to interact with miRNAs involved in cell cycle regulation—in murine fertilized oocytes as a consequence of its paternal transfer to the zygote (Ragusa *et al.*, 2019).

Several physio-pathological conditions drastically change sperm circRNA cargo. Indeed, spermatozoa collected from asthenozoospermic patients showed a deregulated enrichment of circRNAs mainly involved in signalling pathways linked to sperm motility (Manfrevola *et al.*, 2020). Not least, *in vivo* studies carried out in male mice fed with a high-fat diet have demonstrated a functional correlation between lifestyle stressors and sperm circRNA cargo, as well as deranged effects on the ability of sperm to circularize mRNAs, dependent on the aberrant FUS content (Manfrevola *et al.*, 2023).

Despite the consolidated role of circRNAs in the control of male reproductive functions, an alleged functional link between ECs and sperm circRNA cargo has never been investigated. To shed light on this topic, in the current work we decided to analyse the high-quality sperm population collected from normozoospermic men living in a vast geographical area in Campania (Italy), coined as ‘Land of Fires’ and associated with an excessive discharge of toxic contaminants into the environment. This area, located at the North of Naples city, is known for the accumulation of heavy metals and toxic chemicals in the environment (Lettieri *et al.*, 2020a; Alberti 2022). As a consequence, a negative impact on human health, including male reproductive skills, has been recorded in recent years (Fattore *et al.*, 2006; Fattore *et al.*, 2008; Bergamo *et al.*, 2016; Esposito *et al.*, 2017).

Therefore, focusing on Land of Fires as an optimal study case to assess EC-dependent effects on sperm circRNAs, we first carried out morphological and molecular analyses on high-quality spermatozoa collected from young men living in Land of Fires (LF group) or not (CTRL group). The rationale for turning the lens on high-quality spermatozoa for our investigations was to evaluate whether ECs could affect sperm quality and the circRNA cargo of men who, despite being classified as normozoospermic on the basis of morpho-functional parameters, could produce spermatozoa with a poor epigenetic landscape.

Therefore, this study leverages a limited geographic area, namely the Land of Fires, to expand knowledge concerning the catastrophic impact of the environmental pollution on sperm quality, also strengthening the potential role of circRNAs as meaningful determinants in the identification of an epigenetically normal male gamete.

## Materials and methods

### Ethical approval and recruitment

The sperm samples were obtained from men (aged between 25 and 40 years) attending the Infertility Center of Marcianise (Caserta, Italy), together with their female partners, to undergo the initial screening for infertility evaluation, given their failure to achieve a full-term pregnancy. After obtaining their written informed consent, in accordance with the Declaration of Helsinki, we only considered men who, following a routine sperm motility test, were classified as ‘normozoospermic’ and whose problems were related to an ascertained female-factor-associated infertility.

The recruitment of normozoospermic men was carried out in two different geographic areas: participants who resided in municipalities of Land of Fires (belonging to the provinces of Caserta) with the highest illegal discharge of toxic waste (LF group, n = 80) or who resided in the Campania area with low environmental contamination (CTRL group, n = 80). All men were interviewed to better understand their area of origin, their eating habits, as well as their lifestyles. To avoid any experimental interferences, we included in the study subjects who were not exposed to factors potentially affecting semen quality such as chronic and systemic diseases, physio-pathological conditions such as varicocele, and drug abuse. This study involving human participants was reviewed and approved by the ethics committee of Azienda Sanitaria Locale (ASL) Caserta, Regione Campania (ASL CE Prot. N. 1217885/DIR. GE).

### Isolation of high-quality spermatozoa and evaluation of semen parameters

Semen samples were produced by masturbation following 5–7 days of sexual abstinence and collected in sterile containers. After liquefaction for 30 min at 37°C, in accordance with the World Health Organization (2010) reference criteria, semen parameters [volume of semen, pH, sperm cell count, sperm motility (total and progressive), curve-linear velocity, straight-line velocity, average path velocity, linearity, straightness, amplitude of lateral head displacement, and beat-cross frequency], were assessed by using a computer-aided sperm analyzer (CASA), the Sperm Class Analyzer (SCA) system (SCA version 6.1; Microptic, S.L. Viladomat, Barcelona, Spain). The high-quality sperm fraction was purified and separated from the low-quality sperm sub-population using a density gradient centrifugation approach, as previously reported (Chioccarelli *et al.*, 2019). In brief, semen was loaded at the top of a 40/80% discontinuous PureCeption (Cooper Surgical, Trumbull, CT, USA) gradient and centrifuged at 300 × *g* for 20 min. Following centrifugation, seminal plasma was collected in a new tube and subjected to an additional centrifugation step at 21 000 × *g* to allow precipitation of cells and debris. To exclude cellular contamination, seminal plasma supernatant was analysed under a light microscope (Leica CTR500, Leica Microsystems Inc., Milan, Italy), aliquoted and stored at −80°C for biochemical investigations. The high-quality sperm fraction was purified from 80% PureCeption, washed with sperm washing medium (HTF-IrvineScientific^®^) to remove the PureCeption and centrifuged at 500 × *g* for 15 min. After that, sperm samples were treated with somatic cell lysis buffer (SCLB) (0.1% SDS, 0.5% Triton X-100 in DEPC-H_2_O) for 30 min in ice to eliminate any somatic cell contamination. After the SCLB treatment, by using microscope examination, an aliquot of sample was used to verify the elimination of somatic cells and to evaluate the number of live spermatozoa to exclude any dead cell contamination by using the viable dye Trypan-blue (0.4% solution, 17-942E, Lonza, Basel, Switzerland) (data not shown). In addition, CASA-based technology was repeated on the purified sperm fraction to confirm that the density gradient procedure did not change motility and morphological sperm parameters. Lastly, high-quality spermatozoa from both experimental groups were counted under a light microscope to pellet equal concentrations of sperm cells (1 × 10^7^ cells) and stored at −80°C for molecular investigations. Several aliquots of high-quality spermatozoa from both experimental groups were dried on slides and stored at −20°C for morphological and immunofluorescence analysis.

### Chemical element dosage in human seminal plasma

Aliquots of CTRL- and LF-seminal plasma (500 µl; n = 10 for each experimental group) were used for the dosage of 21 elements namely: aluminium (Al), arsenic (As), barium (Ba), beryllium (Be), cadmium (Cd), cobalt (Co), chromium (Cr), copper (Cu), iron (Fe), mercury (Hg), lithium (Li), manganese (Mn), molybdenum (Mo), nickel (Ni), lead (Pb), antimony (Sb), selenium (Se), tin (Sn), thallium (Tl), vanadium (V), and zinc (Zn). The analysis was carried out by a specialized screening service [Proteomic and Metabolomic Laboratory; Consorzio Interuniversitario INBB (Istituto Nazionale Biostrutture e Biosistemi); Naples, Italy]. Element content was calculated by using standard curves and the final concentrations were expressed as µg/l (mean value ± SEM).

### Determination of steroid hormone and dioxin levels in human seminal plasma

#### Materials

2,3,7,8-Tetrachlorodibenzo-*P*-dioxin (TCDD), beta-estradiol (E_2_), progesterone (PG), and testosterone (TT) were procured from Merck (Darmstadt, Germany). MS-grade reagents, including ultrapure water, methanol (MeOH), and acetonitrile (ACN) were acquired from Romil (ROMIL Ltd, Cambridge, UK).

#### Steroid hormone extraction

Following the procedure previously optimised and validated (Errico *et al.*, 2019), steroid hormone extraction from human seminal plasma of CTRL (n = 10) and LF (n = 10) men was performed in two steps: the liquid-liquid extraction of TT, PG, and E_2_ with MeOH followed by the solid phase extraction (SPE) using AFFINIMIP^®^ SPE cartridges (Polyntell SA, Paris, France). Therefore, all seminal plasma samples were centrifuged at 500 × *g* for 30 sec at room temperature to ensure their homogeneity. Then, 500 µl of seminal plasma was mixed with 2 ml MeOH and centrifuged 10 min at room temperature. The supernatant was recovered, mixed with 2 ml H_2_O and then applied to an AFFINIMIP SPE cartridge, previously conditioned with 1 ml ACN and 1 ml H_2_O, following the manufacturer’s instructions. After the sample loading, the cartridge was eluted with 1 ml MeOH. The eluate was collected and dried under stream of nitrogen up to a final volume of ∼0.250 ml. The sample was diluted to 500 µl final volume with H_2_O. Finally, 100 μl of the sample extract was injected for liquid chromatography–electrospray ionization tandem mass spectrometry analysis (LC/ESI-MS/MS) analysis.

#### LC/ESI-MS/MS analysis

A Dionex UltiMate 3000 HPLC system (Thermo Fisher Scientific Inc, Monza, Italy) was used in this study. The chromatographic separation was performed using a 100 mm × 3.0 mm Kinetex 2.6 μm F5 stainless steel HPLC column (Phenomenex, Bologna, Italy). Mobile phase A was water and mobile phase B was MeOH, both without additives such as formic acid, acetic acid, or ammonium acetate. Chromatography was run at room temperature by linear gradient elution with a total run of 13 min. The percentage of B solvent (MeOH) changed as follows: the analysis begins with 30% (v/v) for 1 min, then followed by a gradient from 30% (v/v) to 95% (v/v) in 5 min holding at 95% (v/v) for 4 min. Finally, the mobile phase was decreased to 30% (v/v) in 1 min and equilibrated at 30% (v/v) for further 2 min. The flow rate was 0.3 ml/min. The injection volume was 100 µl.

The HPLC system was coupled to a triple quadrupole mass spectrometer instrument (Sciex, Milano, Italy) equipped with a Turbo-ion electrospray source (ESI-MS/MS). Analytes were detected in both negative and positive ionization modes at a vaporization temperature of 350°C and an ion electrospray voltage (IS) of 5.5/−4.5 kV. The analytes were quantified in multiple reaction monitoring (MRM) mode. The transitions monitored in MRM mode for TT were 291.3 > 97.2 and 291.3 > 108.9 *m*/*z* for its quantifier and qualifier ions, respectively. The transitions monitored in MRM mode for PG were 318.0 > 97.1 and 318.0 > 109.1 *m*/*z* for its quantifier and qualifier ions, respectively. The transitions monitored in MRM mode for E_2_ were 271.2 > 145.1 and 271.2 > 183.2 *m*/*z* for its quantifier and qualifier ions, respectively.

TT, PG, and E_2_ identification was based on the retention time of both quantifier and qualifier product ions. The data were analysed by the Analyst™ software (Sciex, Milano, Italy).

#### Determination of TCDD

Aliquots from human seminal plasma of CTRL (n = 10) and LF (n = 10) men were extracted twice with 2 ml n-hexane and sulphuric acid. The combined hexane layers were cleaned using SPE silica columns (Thermo Fisher Scientific, Waltham, MA, USA). The samples were eluted with 1 ml of ethanol/toluene (70:30), dried under a nitrogen stream, and then reconstituted with 50 μl of toluene. Aliquots (1 μl) of the extracts were introduced into a gas chromatograph coupled with mass spectrometry GC-MS (Thermo Fisher Scientific, Waltham, MA, USA) and analysed using a silica capillary column (25 m × 0.25 mm × 0.25 μm). Pure helium was used as the carrier gas at 1.0 ml/min. Mass spectrometer operates in negative EI mode at 60 eV with a source temperature of 270°C.

### *In vitro* treatment of human high-quality spermatozoa with lead

Lead acetate (Pb) was obtained from Sigma-Aldrich (316512; Milan, Italy) and dissolved in dimethylsulphoxide (DMSO) according to the manufacturer's instructions.

Human high-quality spermatozoa pellets (1 × 10^7^ cells) collected from CTRL normozoospermic men (n = 10) were purified as described above and incubated in PBS (1 ml) for 30 min at 37°C with vehicle [0.005% DMSO; control group (Ctrl)] or with Pb at 10 μM [experimental groups (Pb)]. Dose and incubation time were chosen in order to modulate sperm morpho-functional parameters and acrosome state without affecting cell viability, on the base of previous data (Castellanos *et al.*, 2013; He *et al.*, 2016). In addition, the viable dye Trypan-blue (0.4% solution, 17-942E, Lonza, Basel, Switzerland) was used to evaluate the number of live spermatozoa under a light microscope in order to confirm the absence of harmful effect on cell viability dependent on the treatment (data not shown).

Following *in vitro* treatment, sperm samples were centrifuged at 1500 × *g* for 20 min at 4°C and washed twice with PBS. Sperm pellet was properly stored at −80°C for molecular investigations while several aliquots of sperm samples were dried on slides and stored at −20°C for morpho-functional and immunofluorescence analysis.

### Haematoxylin and eosin staining of human high-quality spermatozoa

Human high-quality spermatozoa collected from CTRL- and LF-normozoospermic men (CTRL-spermatozoa and LF-spermatozoa, respectively; n = 10 for each experimental group) were dried on slides and processed for haematoxylin and eosin (H&E) staining using ordinary procedures. In detail, sperm slides were observed under a light microscope (Leica CTR500; Leica Microsystems Inc., Milan, Italy) and the images were captured using a high-resolution digital camera (Leica DC300F). The number of spermatozoa with an anomalous head morphology was quantified performing a cell counting assay. For each assay, a minimum of 100 sperm cells was counted (n = 10 different samples in triplicate for each experimental group). Data were reported as the percentage of anomalous sperm heads/total spermatozoa (mean value ± SEM). All the results were validated twice by the same operator.

### Immunofluorescence analysis of human high-quality spermatozoa

Aliquots (n = 6 for each experimental group) of spermatozoa collected from CTRL (n = 10) and LF (n = 10) men and *in vitro* treated Ctrl- and Pb-spermatozoa were dried on slides, fixed in 4% paraformaldehyde (sc-281692; Santa Cruz Biotechnology, Heidelberg, Germany) for 20 min at room temperature and permeabilized with 0.1% Triton X-100 (X100; Sigma-Aldrich, Milano, Italy). A 10% donkey serum (ab7475; Abcam, Cambridge, UK) was used to carry out blocking for 30 min at room temperature. Then, the cells were incubated overnight (O.N.) at 4°C with different primary antibodies, all diluted 1:100 [IZUMO1 (ab211623; Abcam, Cambridge, UK); FUS (PA5-52610; Invitrogen, Milano, Italy) and p-300 (ab275378; Abcam, Cambridge, UK)] and, following three washes in Dulbecco's PBS (DPBS, 1×), a FITC (711-095-152) or Cy5 (111-175-144) conjugate was used as secondary antibody, diluted 1:100 (Jackson ImmunoResearch, Cambridge, UK) for 1 h at 37°C. Nuclei were labelled with DAPI (D9542; Sigma-Aldrich, Milano, Italy), and all samples were analysed under an optical microscope (Leica DM 5000 B + CTR 5000; Leica Microsystems, Wetzlar, Germany) with a UV lamp. Images were viewed with IM 1000 software (version 4.7.0; Leica Microsystems, Wetzlar, Germany) and captured by using Leica DFC320 R2 digital camera. To quantify the number of IZUMO1 positive cells, a minimum of 100 sperm cells were counted (n = 10 different samples in triplicate for each experimental group) for each assay. Data were reported as the percentage of IZUMO1-positive cells (mean value ± SEM). All the results were validated twice by the same operator. Densitometric analysis of IZUMO1 immunofluorescence signal (n = 6 for each experimental group) was performed with ImageJ Software (version 1.53 g GISA Elettronica, Naples, Italy) and adjusted relative to DAPI fluorescence intensity. For the fluorescent signal analysis, 20 fields/sample were analysed.

### PNA staining of the acrosome in human high-quality spermatozoa

Aliquots (n = 6 for each experimental group) of spermatozoa collected from CTRL (n = 10) and LF (n = 10) men and *in vitro* treated Ctrl- and Pb-spermatozoa were dried on slides and fixed in 4% paraformaldehyde (sc-281692; Santa Cruz Biotechnology, Heidelberg, Germany) for 20 min at room temperature. Following permeabilization with 0.1% Triton X-100 (X100; Sigma-Aldrich, Milano, Italy), slides were stained with peanut agglutinin (PNA) lectin (L32458; Alexa Fluor 568, Thermo Fisher Scientific, Waltham, MA, USA), diluted 1:50, for 1 h at 37°C to assess acrosome state. Following three washes in DPBS (1×), nuclei were labelled with DAPI (D9542; Sigma-Aldrich, Milano, Italy) and all samples were analysed under an optical microscope (Leica DM 5000 B + CTR 5000; Leica Microsystems, Wetzlar, Germany) with a UV lamp as reported above. To quantify the number of PNA positive cells, a minimum of 100 sperm cells was counted (n = 10 different samples in triplicate for each experimental group) for each assay. Data were reported as the percentage of PNA positive cells (mean value ± SEM). All the results were validated twice by the same operator.

### Immunofluorescence analysis of oxidative stress markers in human high-quality spermatozoa

Aliquots (n = 6 for each experimental group) of spermatozoa collected from CTRL (n = 10) and LF (n = 10) men and *in vitro* treated Ctrl- and Pb-spermatozoa were dried on slides, fixed and permeabilized as reported above. Following blocking, sperm cells were incubated with different primary antibodies diluted 1:100 [3-NT (ab61392; Abcam, Cambridge, UK) and anti-4HNE (ab46545; Abcam, Cambridge, UK)] O.N. at 4°C. Then, three washes in DPBS (1X) were carried out and a FITC (711-095-152) conjugate, diluted 1:100, was used as secondary antibody (Jackson ImmunoResearch, Cambridge, UK) for 1 h at 37°C. Nuclei were labelled with DAPI (D9542; Sigma-Aldrich, Milano, Italy) and all samples were analysed under an optical microscope (Leica DM 5000 B + CTR 5000; Leica Microsystems, Wetzlar, Germany) with a UV lamp as reported above.

### Immunofluorescence analysis of lipid state of sperm plasma membrane

Aliquots (n = 6 for each experimental group) of spermatozoa collected from CTRL (n = 10) and LF (n = 10) men and *in vitro* treated Ctrl- and Pb-spermatozoa were treated as previously reported. After the permeabilization and blocking steps, to analyse the cholesterol complexes, sperm cells were stained with Filipin III at final concentration of 50 μg/ml for 2 h, while to investigate the membrane lipid raft organization, sperm cells were incubated with Flotillin-1 primary antibody diluted 1:100 (sc-74566; Santa Cruz Biotechnology, Heidelberg, Germany) O.N. at 4°C and then with a FITC (711-095-152) conjugated secondary antibody (Jackson ImmunoResearch, Cambridge, UK) diluted 1:100 for 1 h at 37°C. Nuclei were labelled with DAPI (D9542; Sigma-Aldrich, Milano, Italy). For both assays, the slides were analysed under an optical microscope (Leica DM 5000 B + CTR 5000; Leica Microsystems, Wetzlar, Germany) with a UV lamp.

### Sperm motility assay

Aliquots of *in vitro* treated Ctrl- and Pb-spermatozoa (n = 10 for the experimental group) were used to carry out sperm motility assay (in triplicate). The number of viable spermatozoa, assessed through the dye Trypan-blue reagent (0.4% solution, 17-942E, Lonza, Basel, Switzerland), was used to define the percentage of motile/live spermatozoa by two observers using a light microscope (Leica CTR500, Leica Microsystems Inc., Milan, Italy) at a magnification of 20× using a haemocytometer (Burker chamber). For each assay, a minimum of 100 sperm cells was evaluated and counted for each analysis.

### Protein extraction and Western blot analysis

RIPA buffer [PBS, pH 7.4, 10 mM dithiothreitol, 0.02% sodium azide, 0.1% SDS, 1% NP-40, 0.5% sodium deoxycholate, protease inhibitors (10 μg/ml of leupeptin, aprotinin, pepstatin A, chymostatin, and 5 μg/ml of TPCK)] was used to obtain total protein lysates from aliquots (n = 10) of spermatozoa collected from CTRL (n = 10) and LF (n = 10) men. Then, proteins were separated by sodium dodecyl sulphate-polyacrylamide gel electrophoresis (SDS-PAGE) (4–20% Mini-PROTEAN^®^ TGX™ Precast Protein Gels; 4561094, Bio-Rad Laboratories, Milano, Italy) and transferred to polyvinylidene difluoride membrane (GE Healthcare, Milano, Italy) at 280 mA for 2.5 h at 4°C. Following blocking [5% non-fat milk, 0.25% Tween-20 in Tris-buffered saline (TBS, pH 7.6)], the filters were incubated with different primary antibodies, all diluted 1:1000 [IZUMO1 (ab211623) and tubulin (ab15246) from Abcam, Cambridge, UK]. After washing in Tween20-TBS, filters were incubated with 1:1000 horseradish peroxidase-conjugated rabbit IgG (Dako Corp., Milano, Italy) in TBS-milk buffer. An enhanced chemiluminescence-western blotting detection system [Amersham ECL western Blotting Detection Reagent (RPN2106) GE Healthcare, Milano, Italy] was used to detect the immune complexes. Densitometry analysis of signals was carried out relatively to tubulin and presented as optical density fold change (mean ± SEM).

### Protein immunoprecipitation

Aliquots (n = 6 for each experimental group) of spermatozoa collected from CTRL (n = 10) and LF (n = 10) men and *in vitro* treated Ctrl- and Pb-spermatozoa were used for immunoprecipitation (IP) experiments. In detail, a concentration of 500 mg total protein lysate from each experimental group was incubated with 2 μg of relative antibody: FUS antibody (PA5-52610; Invitrogen, Milano, Italy) or IgG (12370; Sigma-Aldrich, Milano, Italy) under rotary agitation O.N. at 4°C. Protein A/G PLUS Agarose Beads (sc-2003; Santa Cruz Biotechnology, Heidelberg, Germany) were then added to each sample for 4 h, at 4°C under rotary agitation. After three washes in 500 µl of cold TBS pH 7.6 (3000 × *g* for 3 min a 4°C), all samples were boiled in Laemmli sample buffer for 10 min to be later analysed by SDS-PAGE in comparison to related input controls.

### Total RNA preparation

Aliquots (n = 10 for each experimental group) of spermatozoa collected from CTRL (n = 10) and LF (n = 10) men and *in vitro* treated Ctrl- and Pb-spermatozoa were used to extract total RNA using TRIzol Reagent (Invitrogen Life Technologies, Paisley, UK) following the manufacturer’s instructions. After sample homogenization (1 ml TRIzol Reagent/5–10 × 10^6^ sperm cells) for 5 min at 20°C, 0.2 ml chloroform/ml TRIzol Reagent were added to each sample and centrifuged at 12 000 × *g* for 15 min at 4°C. The addition of isopropyl alcohol (0.5 ml/ml TRIzol Reagent) and 1 µl glycogen (20 mg/ml) to the aqueous phase ensured total RNA precipitation. Each RNA pellet was washed with 75% ethanol, centrifuged at 7500 × *g* for 10 min at 4°C, and dissolved in DEPC-treated water to quantify (ng/µl) and evaluate RNA purity (260/280 and 260/230 ratios) using a NanoDrop 2000 spectrophotometer (Thermo Fisher Scientific, Waltham, MA, USA). Aliquots of RNA (10 µg) were treated with 2U DNase I (RNase-free DNase I, Ambion, Thermo Fisher Scientific, Waltham, MA, USA) to remove genomic DNA contamination. The RNAs were then stored at −80°C until the next molecular investigations.

### RNA expression analysis by one-step quantitative RT-PCR

CircRNA expression analysis in spermatozoa collected from CTRL and LF men and in *in vitro* treated Ctrl- and Pb-spermatozoa was carried out on a CFX-96 Real Time PCR System (Bio-Rad, Milano, Italy) using the One-Step Evagreen quantitative RT-PCR (qRT-PCR) reaction kit containing a qRT-PCR enzyme mix and an Evagreen qPCR Mastermix (Applied Biological Materials Inc., Ferndale, WA, USA). Each assay was carried out in triplicate and included a negative control, without RNA, and the melting curve analysis of primer pairs. CFX Manager software (Bio-Rad, Milano, Italy) was used for RNA expression analysis. *GAPDH* was chosen as housekeeping gene for data normalization. The normalised fold expression (nfe) of circRNAs was calculated by applying the 2^−ΔΔCt^ method. All the results were expressed as a mean value of nfe ± SEM.

### RNA-binding protein immunoprecipitation assay

Aliquots (n = 6 for each experimental group) of: i) spermatozoa collected from CTRL (n = 10) and LF (n = 10) men and ii) *in vitro* treated Ctrl- and Pb-spermatozoa were used for RNA-binding protein immunoprecipitation (RIP) experiments. Sperm samples were lysed in RIPA buffer to obtain total protein samples, as reported above. An equal concentration of protein lysates was incubated with 5 µg of FUS antibody (PA5-52610; Invitrogen, Milano, Italy) or IgG (12370; Sigma-Aldrich, Milano, Italy) under rotary agitation at 4°C. Then, for each sample, an appropriate volume (50 µl) of protein A/G PLUS agarose beads (sc-2003; Santa Cruz Biotechnology, Heidelberg, Germany) was incubated for 4 h at 4°C. The resulting bead pellets were used to carry out total RNA extraction using TRIzol Reagent (Invitrogen Life Technologies, Paisley, UK). RNAs immunoprecipitated with FUS and control IgG antibodies were quantified by NanoDrop 2000 spectrophotometer (Thermo Fisher Scientific, Waltham, MA, USA) and stored at −80°C until circRNA qRT-PCR analysis.

### PCR primer design

The primers to amplify circRNAs of interest were designed by the online tool Primer-BLAST (http://www.ncbi.nlm.nih.gov/tools/primer-blast/, accessed on 15 February 2021) in order to span the backsplicing junction specific for the circular isoforms. Primer sequences are shown in Table 1.

**Table 1.** Primer sequences and annealing temperatures used in quantitative RT-PCR.

Gene name	Gene primers	Sequences 5′–3′	Tm (°C)
*CCR4-NOT Transcription Complex Subunit 6 Like*	*circCNOT6L S*	GCCTTATGAACTTGGTCGGCT	56
	*circCNOT6L* AS	TTCTGCGAGGATCTGGAGGAT	
*Leucine Zipper and CTNNBIP1 Domain Containing*	*circLZIC* S	ACAGACCTTGGCTATTCAGGC	56
	*circLZIC* AS	AACCTTGTCCGAAGCTGACC	
*L3MBTL Histone Methyl-Lysine Binding Protein 4*	*circL3MBTL4* S	GGTCTTGGGAGTGGTACTTGA	58
	*circL3MBTL4* AS	CCTTGGCAGTGGTTTTATGGTC	
*RAS P21 Protein Activator 3*	*circRASA3* S	GCAGAAGTACCACAACAGGGA	56
	*circRASA3* AS	TTTGGCTTCACCTGCACTTC	
*Argonaute RISC Catalytic Component 2*	*circEIF2C2* S	CGGGATCACCTTCATCGTGG	58
	*circEIF2C2* AS	GTATGATCTCCTGCCGGTGC	
*Mitochondrially Encoded NADH: Ubiquinone Oxidoreductase Core Subunit 5*	*circMTND5* S	GCAGCCTAGCATTAGCAGGAAT	56
	*circMTND5* AS	GGGAGGTTGAAGTGAGAGGTA	
	*GAPDH* S	TGCACCACCAACTGCTTAGC	58
	*GAPDH* AS	GGCATGGACTGTGGTCATGAG	

AS: antisense; S: sense; Tm: melting temperature.

### Correlation analysis

Data of sperm circRNA expression (normalized values from independent qRT-PCR analyses) and Pb seminal plasma levels relative to normozoospermic men (n = 20) were correlated with each other. The Shapiro–Wilk test was applied to continuous variables to check for distribution normality. Data were compared by Pearson's correlation analyses using the Excel built-in distribution functions available in Microsoft Office (GISA Elettronica, Naples, Italy). The ‘*r*’ value was considered to establish the test significance. The range −1 ≤ *r* ≤ 1 established negative or positive correlation.

### Statistical analysis

The data normality was assessed utilizing the Shapiro-Wilk test, and following confirmation of normal distribution of data, the Student's *t*-test (for two independent group comparisons) was used to identify groups having different means. Differences with *P* < 0.05 were considered statistically significant. Data were expressed as the mean ± SEM.

For western blot, qRT-PCR, and RIP analyses, triplicates from at least 6/10 different experimental groups were considered statistically significant. For cell counting assays triplicates from 10 different experimental groups were considered statistically significant.

## Results

### ECs accumulate in the seminal plasma and affect semen parameters of normozoospermic men

With the aim to investigate environmental contaminant-dependent effects on male reproductive health, we carried out a systematic study on young men living (LF) or not (CTRL) in Land of Fires. In detail, seminal plasma was collected from CTRL and LF men and used for functional and biochemical investigations.

First, we carried out semen analysis by using CASA technology, in order to include in the study only subjects classified as normozoospermic, thus excluding any interference dependent on pathophysiological conditions. As reported in Table 2, the main semen parameters showed no statistically significative differences between the two experimental groups.

**Table 2.** Semen parameters of healthy normozoospermic men living in the Land of Fires or outside of that area.

Semen parameters	CTRL n = 80	LF n = 80
Volume (ml)	2.7 ± 1.1	2.9 ± 0.9
pH	7.7 ± 0.3	7.8 ± 0.5
Total sperm number (10^6^/ejaculate)	129.5 ± 12.9	126.5 ± 9.7
Cell concentration (10^6^/ml)	37.5 ± 5.9	33.5 ± 3.7
Total motility (%)	89.5 ± 2.7	83.2 ± 3.4
Progressive motility (%)	67.5 ± 2.9	62.2 ± 1.8
VCL (µm/s)	63.5 ± 2.3	57.1 ± 2.6
VSL (µm/s)	36.7 ± 2.8	32.5 ± 2.1
VAP (µm/s)	35.8 ± 1.7	31.3 ± 2.5
LIN (%)	62.6 ± 3.2	58.2 ± 2.9
STR (%)	88.2 ± 2.6	83.1 ± 3.3
ALH (µm)	2.1 ± 1.1	1.9 ± 0.9
BCF (Hz)	8.8 ± 1.3	8.4 ± 1.2

Data are mean ± SEM.

ALH: amplitude of lateral head displacement; BCF: beat-cross frequency; CTRL: control group, men not living in the ‘Land of Fires’; LIN: linearity; LF: the group of men living in the ‘Land of Fires’, which is a large geographical area located in Campania, Italy, where excessive illegal discharge of toxic waste into the environment occurs; STR: straightness; VAP: average path velocity; VCL: curve-linear velocity; VSL: straight-line velocity.

Biochemical dosage of a wide set of chemical elements, including heavy metals, showed a significative reduction of Mn, Zn, Sn, and Ba (*P* < 0.01 or *P* < 0.05) in LF seminal plasma, whereas higher levels of Cd and Pb (*P* < 0.05) were observed in LF when compared with CTRL seminal plasma (Table 3). In addition, increased levels of TCDD (*P* < 0.05) and several dioxin-like PCBs (*P* < 0.05) were detected in LF seminal plasma (Table 4).

**Table 3.** Heavy metal dosage (µg/l) in seminal plasma collected from normozoospermic men.

Metal	CTRL	LF
Li	1.2024 ± 0.050	1.141875 ± 0.050
Be	0.0038 ± 0.003	0.0015 ± 0.002
Al	0.6558 ± 0.045	0.518125 ± 0.063
V	0.2772 ± 0.024	0.1855 ± 0.024
Cr	0.165 ± 0.026	0.2455 ± 0.024
Mn	1.4188 ± 0.021	1.057875 ± 0.034**
Fe	0.842 ± 0.139	0.859375 ± 0.074
Co	0.0454 ± 0.008	0.0785 ± 0.008
Ni	0.2768 ± 0.096	0.34328571 ± 0.074
Cu	0.2506 ± 0.026	0.259625 ± 0.036
Zn	939.8132 ± 0.021	669.007625 ± 0.053*
As	0.2684 ± 0.018	0.25375 ± 0.014
Se	0.1248 ± 0.018	0.15975 ± 0.019
Mo	0.373 ± 0.056	0.349375 ± 0.057
Cd	0.0545 ± 0.010	0.167 ± 0.022*
Sn	0.21425 ± 0.004	0.073375 ± 0.015*
Sb	0.0332 ± 0.022	0.048 ± 0.038
Ba	1.72925 ± 0.092	0.873125 ± 0.024**
Tl	0.001 ± 0.006	0.00125 ± 0.003
Pb	1.0605 ± 0.018	1.348375 ± 0.021**
Hg	0.0124 ± 0.002	0.01675 ± 0.002

CTRL n = 10; LF n = 10; data are mean± SEM.

* *P* < 0.05.

** *P* < 0.01.

CTRL: control group, men not living in the Land of Fires; LF: the group of men living in the ‘Land of Fires’, which is a large geographical area located in Campania, Italy, where excessive illegal discharge of toxic waste into the environment occurs.

**Table 4.** Dioxin dosage (pg/ml) in seminal plasma collected from CTRL- and LF-normozoospermic men.

Dioxins	CTRL	LF
TCDD	8.143 ± 0.211	17.500 ± 0.342*
PCB-28	1.256 ± 0.098	3.504 ± 0.104
PCB-52	1.844 ± 0.430	2.375 ± 0.568
PCB-101	2.067 ± 0.014	3.879 ± 0.010*
PCB-118	2.088 ± 0.056	2.783 ± 0.078
PCB-138	7.785 ± 0.311	9.001 ± 0.498
PCB-153	3.589 ± 0.012	7.913 ± 0.008*
PCB-180	3.967 ± 0.004	7.913 ± 0.005*
∑ PCB	21.050 ± 0.012	37.640 ± 0.009*

CTRL n = 10; LF n = 10; data are mean± SEM.

CTRL: control group, not living in the Land of Fires; LF: the group of men living in the ‘Land of Fires’, which is a large geographical area located in Campania, Italy, where excessive illegal discharge of toxic waste into the environment occurs; PCB: polychlorinated biphenyls; TCDD: 2,3,7,8-tetrachlorodibenzo-*P*-dioxin.

* *P* < 0.05.

### ECs affect sperm morpho-functional parameters of high-quality spermatozoa

**Figure 1.**
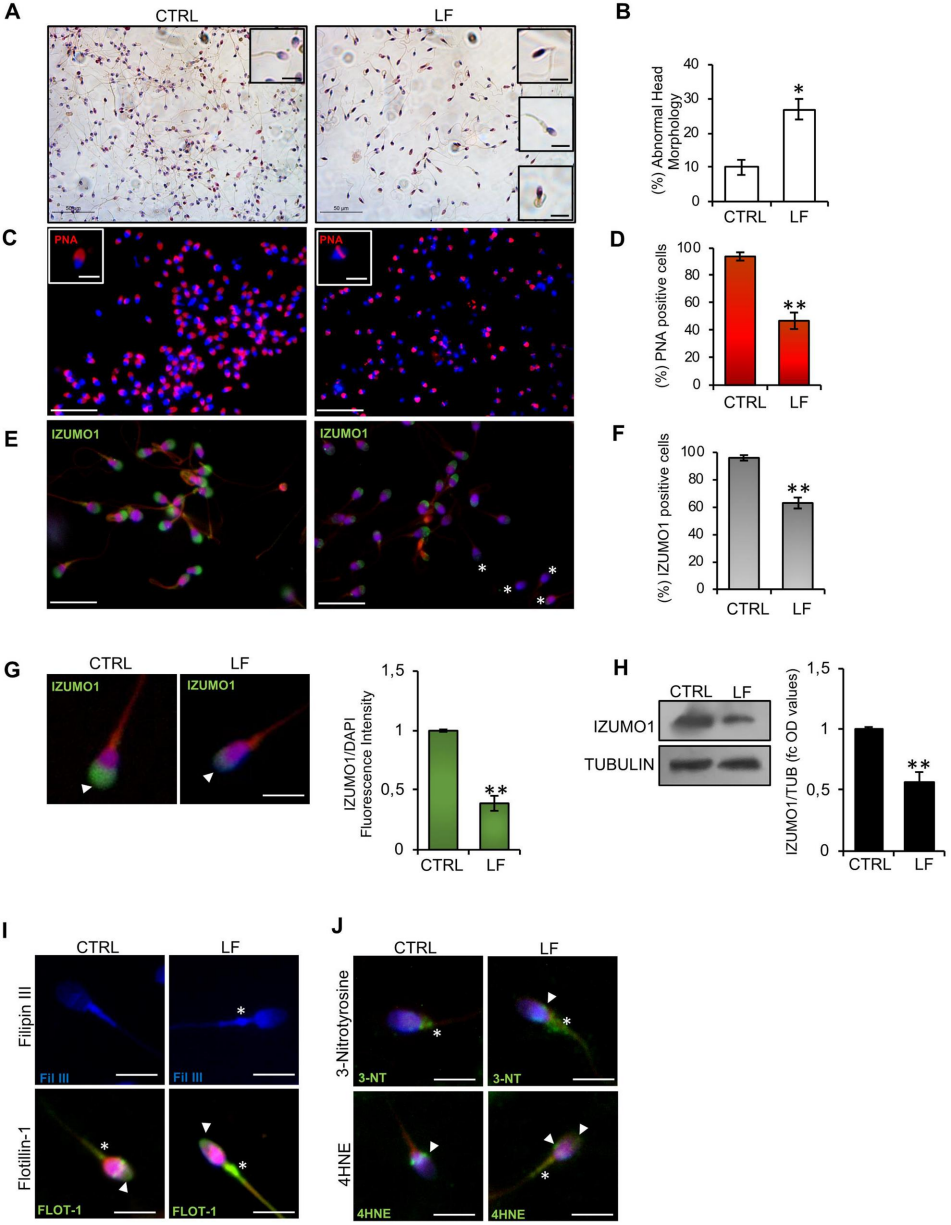
**Morpho-functional characterization of high-quality spermatozoa in normozoospermic young men.** (**A**) H&E staining of high-quality CTRL- and LF-spermatozoa (n = 10 for each experimental group); scale bar: 50 μm. Physiological and anomalous sperm head morphology is indicated in the insets; scale bar inset: 10 μm. (**B**) Cell count of sperm cells having anomalous head morphology in high-quality CTRL- and LF-spermatozoa (n = 10 different samples in triplicate for each experimental group). Data are expressed as the percentage of anomalous sperm heads/total spermatozoa and reported as mean ± SEM; *P < 0.05. (**C**) Immunofluorescence analysis of PNA (red) in high-quality CTRL- and LF-spermatozoa (n = 6 different samples in triplicate for each experimental group). Nuclei were labelled with DAPI (blue). Scale bar: 50 μm; scale bar inset: 5 μm. (**D**) Cell count of PNA positive sperm cells in high-quality CTRL- and LF-spermatozoa (n = 6 different samples in triplicate for each experimental group). Data are expressed as percentage of positive cells on total and reported as mean ± SEM; **P < 0.01. (**E**) Immunofluorescence analysis of IZUMO1 (FITC-green) in high-quality CTRL- and LF-spermatozoa (n = 6 different samples in triplicate for each experimental group). Nuclei were labelled with DAPI (blue); white asterisks represent negative IZUMO1 sperm cells. Scale bar: 20 μm. (**F**) Cell count of IZUMO1-positive sperm cells in high-quality CTRL- and LF-spermatozoa (n = 6 different samples in triplicate for each experimental group). Data are expressed as percentage of positive cells on the total and reported as mean ± SEM; **P < 0.01. (**G**) Immunofluorescence analysis and histogram showing the quantification of IZUMO1 signal intensity in high-quality CTRL- and LF-spermatozoa (n = 6 different samples in triplicate for each experimental group). Values are expressed as mean ± SEM; **P < 0.01; scale bar: 4 μm. (**H**) Western blot analysis of IZUMO1 in high-quality CTRL- and LF-spermatozoa (n = 10 different samples in triplicate for each experimental group). Signals were quantified by densitometry analysis and normalized to tubulin. Data are expressed in OD values as fold change and reported as mean ± SEM; **P < 0.01. (**I**) Filipin III staining and Flotillin-1 (FITC-green) immunofluorescence analyses in high-quality CTRL- and LF-spermatozoa (n = 6 different samples for each experimental group). White arrowheads represent sperm head localizations; white asterisks represent sperm midpiece localizations. Nuclei were labelled with DAPI (blue); scale bar: 4 μm. (**J**) 3-NT (FITC-green) and 4HNE immunofluorescence analyses in high-quality CTRL- and LF-spermatozoa (n = 6 different samples for each experimental group). White arrowheads represent sperm head and neck localizations; white asterisks represent sperm midpiece localizations. Nuclei were labelled with DAPI (blue); scale bar: 4 μm. CTRL: control group, men not living in the Land of Fires; H&E: haematoxylin and eosin; 4HNE: 4-hydroxy-2-nonenal; LF: the group of men living in the ‘Land of Fires’, which is a large geographical area located in Campania, Italy, where excessive illegal discharge of toxic waste into the environment occurs; OD: optical density; PNA: peanut agglutinin. Applied statistical test: Student's *t*-test.

Considering the high bioaccumulation of toxic contaminants in the seminal plasma of LF men, we isolated high-quality spermatozoa from CTRL- and LF-normozoospermic men in order to assess the effective morpho-functional quality of this sperm population. Morphological characterization carried out by H&E staining highlighted several anomalies in high-quality spermatozoa collected from LF subjects, in terms of head shaping and tail structure defects, typically related to a teratozoospermic phenotype (Fig. 1A). In particular, LF-spermatozoa showed head abnormalities, such as microcephalous, thin and tapered heads associated with tail defects, including bent or coiled tails (Fig. 1A insets). In addition, an abnormal residual cytoplasm was observed at midpiece level (Fig. 1A insets). Accordingly, a significant increase (*P* < 0.05) in the percentage of sperm cells with abnormal head morphology was observed among high-quality spermatozoa collected from LF in comparison with the CTRL group (Fig. 1B). Then, the acrosome morphology was evaluated by immunofluorescence analysis using peanut lectin PNA antibody. As shown (Fig. 1C), in high-quality spermatozoa from CTRL subjects, the PNA signal was well confined to the half anterior part of sperm head consisting of the acrosomal region (Fig. 1C inset). Conversely, in the LF experimental group, a high frequency of spermatozoa showing PNA null signal or residual signal localized in the post-acrosomal region was observed (Fig. 1C inset). In agreement, relative to CTRL group, sperm count using acrosomal PNA localization as the inclusive analysis parameter showed a significant reduction (*P* < 0.01) in the percentage of sperm cells with positive PNA signal in the LF group (Fig. 1D), thus suggesting a potential early acrosome reaction (AR) onset. With this in mind, we investigated the localization of IZUMO1, a membrane glycoprotein confined to the acrosome and mainly supporting sperm-oocyte interaction (Inoue *et al.*, 2005) by immunofluorescence analysis (Fig. 1E). As reported, in high-quality spermatozoa collected from CTRL, IZUMO1 appeared physiologically localized to the acrosomal region, similar to the PNA staining pattern, whereas IZUMO1 null or reduced, as well as a more diffused, signal was observed in LF experimental group (Fig. 1E). Interestingly, the sperm count using acrosomal IZUMO1 localization as the morphological parameter of good sperm quality showed a significant reduction (*P* < 0.01) in the percentage of IZUMO1 positive cells in the LF group, similar to the PNA trend (Fig. 1F). In addition, fluorescence intensity analysis confirmed a significant (*P* < 0.01) downregulation of IZUMO1 signal in LF- when compared with CTRL-spermatozoa (Fig. 1G). In accordance with immunofluorescence data, western blot analysis showed a significant reduction (*P* < 0.01) of IZUMO1 protein content in LF-spermatozoa, strongly suggesting the hypothesis that the peculiar PNA and IZUMO1 staining patterns observed in high-quality spermatozoa collected from LF subjects could represent an index of early AR onset.

Steroid hormone levels in the seminal plasma correlate with sperm quality. In particular, high seminal plasma levels of E_2_ correlate with pathozoospermic conditions and seminal TT influences sperm concentration; moreover, PG participates in the regulation of AR (Chen *et al.*, 2019; Calogero *et al.*, 2000; Osadchuk *et al.*, 2023). Based on this evidence, we carried out a biochemical dosage of TT, E_2_, and PG in the seminal plasma collected from CTRL and LF-normozoospermic men. As reported in Table 5, significantly higher levels of E_2_, PG, and TT (*P* < 0.05) were measured in LF than in the CTRL seminal plasma, strongly suggesting a tight relation between hormone levels and sperm morpho-functional anomalies, including putative early AR onset, in LF spermatozoa.

**Table 5.** Hormone dosage (ng/ml) in seminal plasma collected from normozoospermic men.

Hormones	CTRL	LF
17-β-Estradiol (E_2_)	0.442 ± 0.015	21.182 ± 0.121*
Testosterone (TT)	0.113 ± 0.010	0.498 ± 0.023*
Progesterone (PG)	0.072 ± 0.013	0.145 ± 0.034*

CTRL n = 10; LF n = 10; data are mean ± SEM.

CTRL: control group, men not living in the Land of Fires; LF: the group of men living in the ‘Land of Fires’, which is a large geographical area located in Campania, Italy, where excessive illegal discharge of toxic waste into the environment occurs.

* *P* < 0.05.

Lastly, immunofluorescence analyses were performed to evaluate the lipid state of the sperm plasma membrane and the oxidative stress by using selective markers. Cholesterol complexes were analysed in high-quality spermatozoa collected from CTRL- and LF-normozoospermic men by Filipin III staining. Both experimental groups showed a specific Filipin fluorescent signal detectable in cholesterol complexes of the sperm membrane from the head to the tail. Interestingly, a strong signal, specifically localized at the level of sperm head and of the abnormal residual cytoplasm in midpiece region, was detected in LF-spermatozoa (Fig. 1I), suggesting a cholesterol accumulation potentially impacting the membrane fluidity and, therefore, sperm motility. In addition, Flotillin-1 immunofluorescence, chosen as a membrane lipid raft marker expressed in mammalian sperm cells (Maldonado-García *et al.*, 2017), showed a specific signal localized in the half anterior part of the sperm head and in midpiece region, in both experimental groups. In accordance with Filipin III immunofluorescence staining, a higher Flotillin-1 signal, specifically localized at the abnormal residual cytoplasm in midpiece region, was detected in LF- than CTRL-spermatozoa (Fig. 1I), further confirming an abnormal lipid arrangement of sperm plasma membrane. Then we carried out the immunofluorescence analysis of 3-NT, a product derived from ROS-dependent protein tyrosine nitration and used as a marker of sperm oxidative stress (Cruz and Fardilha 2016), and of 4HNE, a product of lipid peroxidation able to impair ATPase activity also used for oxidative state monitoring (Dalleau *et al.*, 2013; Breitzig *et al.*, 2016). Immunofluorescence analysis showed a well-defined signal of both markers proximal to the neck-tail region in CTRL-spermatozoa. Surprisingly, a high and diffused signal throughout the midpiece was observed in LF-spermatozoa (Fig. 1J), suggesting an increased sperm oxidative stress state. Considering that plasma membrane fluidity and ROS production finely regulate sperm motility skills, we performed a detailed sperm motility analysis on high-quality sperm populations isolated from CTRL- and LF-normozoospermic men, using the CASA system. A significant reduction in sperm motility parameters was highlighted in LF- as compared with CTRL-high-quality spermatozoa (*P* < 0.05; *P* < 0.01) (Table 6).

**Table 6.** Motility parameters of high-quality spermatozoa from normozoospermic men.

Motility parameters	CTRL	LF
Total motility (%)	84.1 ± 1.3	72.2 ± 1.8**
Progressive motility (%)	63.7 ± 1.7	54.7 ± 1.5*
VCL (µm/s)	58.2 ± 1.6	44.6 ± 1.2**
VSL (µm/s)	32.5 ± 1.8	25.4 ± 1.5*
VAP (µm/s)	33.1 ± 1.9	24.1 ± 2.1*
LIN (%)	60.5 ± 1.1	51.2 ± 1.3**
STR (%)	86.2 ± 0.9	80.1 ± 1.1*
ALH (µm)	2.0 ± 1.2	1.8 ± 1.3
BCF (Hz)	8.1 ± 1.4	8.3 ± 1.5

CTRL n = 10; LF n = 10; data are mean± SEM.

* *P* < 0.05.

** *P* < 0.01.

ALH: amplitude of lateral head displacement; BCF: beat-cross frequency; CTRL: control group, men not living in the Land of Fires; LIN: linearity; LF: the group of men living in the ‘Land of Fires’, which is a large geographical area located in Campania, Italy, where excessive illegal discharge of toxic waste into the environment occurs; STR: straightness; VAP: average path velocity; VCL: curve-linear velocity; VSL: straight-line velocity.

### ECs affect sperm circRNAs in high-quality spermatozoa

**Figure 2.**
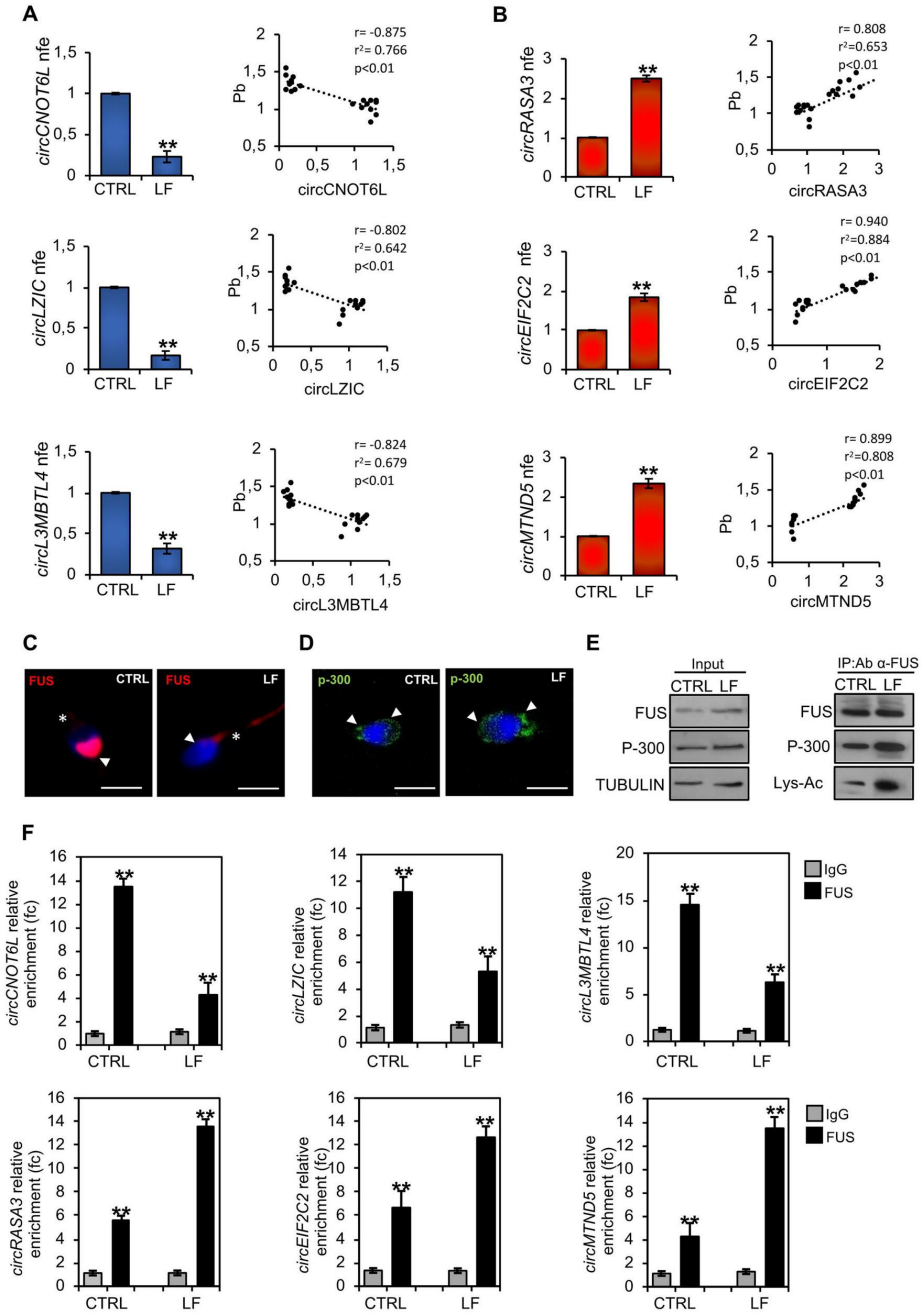
**Environmental contaminants affect FUS-dependent sperm circular RNA backsplicing in sperm from normozoospermic men.** (**A** and **B**) Expression analysis of circRNAs (cirCNOT6L; circLZIC; circL3MBTL4; circRASA3; circEIF2C2; circMTND5) in high-quality CTRL- and LF-spermatozoa (n = 10 different samples in triplicate for each experimental group). qRT-PCR data were normalized using GPDH, expressed as fold expression (nfe) and reported as mean value ± SEM. **P < 0.01. (A and B) Correlation analysis between circRNA expression values and Pb seminal plasma levels relative to high-quality LF-spermatozoa (n = 20). (**C**) Immunofluorescence analysis of FUS (red) in high-quality CTRL- and LF-spermatozoa (n = 6 different samples for each experimental group). Nuclei were labeled with DAPI (blue); white arrowheads represent FUS localization in sperm head; white asterisks represent FUS localization in sperm tail. Scale bar: 4 μm. (**D**) Immunofluorescence analysis of p-300 (FITC-green) in high-quality CTRL- and LF-spermatozoa (n = 6 different samples for each experimental group). Nuclei were labelled with DAPI (blue); white arrowheads represent p-300 localization in sperm head and neck. Scale bar: 4 μm. (**E**) Western blot analysis of protein immunoprecipitation (IP) in high-quality CTRL- and LF-spermatozoa (n = 10 different samples for each experimental group) using FUS antibody. FUS-IP was analysed in comparison with Input protein extracts. (**F**) The enrichment levels of circRNAs (cirCNOT6L; circLZIC; circL3MBTL4; circRASA3; circEIF2C2; circMTND5) in the products of the RIP assay (FUS-IP compared with IgG-IP) in high-quality CTRL- and LF-spermatozoa detected by qRT-PCR. Data are reported as mean ± SEM (n = 6 different samples for each experimental group in triplicate); **P < 0.01. circRNA: circular RNA; CTRL: control group, men not living in the Land of Fires; FUS: fused in sarcoma; LF: the group of men living in the ‘Land of Fires’, which is a large geographical area located in Campania, Italy, where excessive illegal discharge of toxic waste into the environment occurs.; qRT-PCR: quantitative RT-PCR; RIP: RNA-binding protein immunoprecipitation. Applied statistical test: Student's *t*-test.

Our previous findings have defined sperm circRNAs as new epigenetic markers to identify high-quality spermatozoa in terms of structural integrity and motility (Chioccarelli *et al.*, 2019). With this in mind, we decided to investigate a functional correlation between environmental exposure to toxic contaminants and selective circRNAs classified as biomarkers of high- and low-sperm quality (Chioccarelli *et al.*, 2019). We analysed the expression levels of two sets of circRNAs linked to sperm high- (cirCNOT6L; circLZIC; circL3MBTL4) and low- (circRASA3; circEIF2C2; circMTND5) quality parameters, respectively, in high-quality spermatozoa collected from CTRL- and LF-normozoospermic men by qRT-PCR analysis. Interestingly, the content of all circRNAs related to sperm high-quality was significantly decreased (*P* < 0.01) in LF- compared with CTRL-spermatozoa; conversely, the expression levels of circRNAs chosen as sperm low-quality markers were significantly higher (*P* < 0.01) in LF- than in CTRL-spermatozoa (Fig. 2A and B), suggesting that ECs negatively affected the sperm circRNA cargo. In order to strengthen this correlation, we pointed our attention on Pb seminal plasma content a as potential key factor involved in the modulation of sperm circRNA dynamics, as the exposure to Pb severely impairs sperm count, motility, viability, morphology and DNA integrity (Kumar, 2018). Thus, we carried out a correlation analysis between Pb levels in the seminal plasma and the expression data of circRNAs linked to high- or low-sperm quality in CTRL and LF-spermatozoa (Fig. 2A and B). This analysis showed that Pb levels in the seminal plasma were negatively correlated with the expression levels of circRNAs related to high-quality spermatozoa (circCNOT6L: *r* = −0.875, *r*^2^ = 0.766, *P* < 0.01; circLZIC: *r* = 0.802, *r*^2^ = 0.642, *P* < 0.01; circL3MBTL4: *r* = 0.824, *r*^2^ = 0.679, *P* < 0.01) (Fig. 2A) and positively correlated with the expression levels of circRNAs related to low-quality spermatozoa (circRASA3: *r* = 0.808, *r*^2^ = 0.653, *P* < 0.01; circEIF2C2: *r* = 0.940, *r*^2^ = 0.884, *P*< 0.01; circMTND5: *r* = 0.899, *r*^2^ = 0.808, *P*< 0.01) (Fig. 2B).

Interestingly, with the exception of Cd and Zn, whose levels showed a significant but moderate correlation, heavy metals, such as Ba, Sn, and Mn, are significantly (positively or negatively) correlated with the expression data of circRNAs linked to high- or low-sperm quality (Table 7). We chose to focus our attention on Pb for further experiments considering its predominant presence in the industrial and urban waste illegally released in Land of Fires.

**Table 7.** Correlation analysis between circular RNA expression values and seminal plasma metal levels relative to high-quality spermatozoa from men living in the Land of Fires.

Metal	circRNA expression	Pearson’s coefficient correlation (*r*)	*P* value
Cadmium (Cd)	circCNOT6L	−0.50	<0.001
	circLZIC	−0.49	<0.001
	circL3MBTL4	−0.50	<0.001
	circRASA3	0.53	0.016
	circEIF2C2	0.60	<0.001
	circMTND5	0.52	<0.001
Zinc (Zn)	circCNOT6L	0.50	<0.001
	circLZIC	0.46	<0.001
	circL3MBTL4	0.48	<0.001
	circRASA3	−0.38	NS
	circEIF2C2	−0.45	<0.001
	circMTND5	−0.4	<0.001
Selenium (Se)	circCNOT6L	0.77	<0.001
	circLZIC	0.82	<0.001
	circL3MBTL4	0.82	<0.001
	circRASA3	−0.67	0.0012
	circEIF2C2	−0.74	<0.001
	circMTND5	−0.80	<0.001
Manganese (Mn)	circCNOT6L	0.86	<0.001
	circLZIC	0.88	<0.001
	circL3MBTL4	0.89	<0.001
	circRASA3	−0.69	<0.001
	circEIF2C2	−0.80	<0.001
	circMTND5	−0.87	<0.001
Barium (Ba)	circCNOT6L	0.93	<0.001
	circLZIC	0.86	<0.001
	circL3MBTL4	0.87	<0.001
	circRASA3	−0.80	<0.001
	circEIF2C2	−0.87	<0.001
	circMTND5	−0.91	<0.001

N = 20 levels of circRNA expression and of heavy metals.

circRNA: circular RNA.

Based on the evidence that FUS is the main RBP driving circRNA biogenesis in human spermatozoa (Chioccarelli *et al.*, 2021a) and in order to shed light on the anomalous circRNA expression pattern identified in LF-spermatozoa, we investigated FUS protein in CTRL- and LF-spermatozoa by immunofluorescence analysis. As reported in Fig. 2C, in CTRL-spermatozoa FUS protein showed a clear localization in the anterior half part of sperm head and a weak signal in the apical area of the midpiece. Conversely, LF-spermatozoa showed a strong FUS signal in the entire tail length, associated with a weak signal in sperm head (Fig. 2C). In other cellular systems, p-300 acetylase promotes FUS acetylation and, in turn, its mislocalization to the cytoplasm compartment (Arenas *et al.*, 2020). With this background, in order to investigate a similar molecular mechanism in LF-spermatozoa, we performed p-300 immunofluorescence analysis and IP experiments in CTRL and LF-spermatozoa. As shown, p-300 localization was related to the anterior half part of sperm head and to the posterior neck region in both experimental groups (Fig. 2D). Then, we carried out IP using FUS antibody in CTRL and LF-spermatozoa, separately, in order to investigate a possible differential interaction rate among FUS and p-300 acetylase able to abnormally promote the FUS acetylation state in LF-spermatozoa (Fig. 2E). Western blot analysis showed an increase of FUS/p-300 interaction rate in LF-spermatozoa, not dependent on variations of FUS and p-300 protein contents, as confirmed by the analysis of input samples (total lysates isolated before the IP) (Fig. 2E). Interestingly, a significant increase of FUS acetylation was observed in LF-spermatozoa, thus suggesting that an enhanced FUS/p-300 interaction promoted FUS hyperacetylation in LF-spermatozoa, and, in turn, its nucleo-cytosolic shuttling.

Since we previously reported that circRNAs related to high- (cirCNOT6L; circLZIC; circL3MBTL4) and low- (circRASA3; circEIF2C2; circMTND5) sperm quality were preferentially localized in sperm head and tail, respectively (Chioccarelli *et al.*, 2019), we hypothesized that FUS shuttling in sperm tail was responsible for the increase of circRASA3, circEIF2C2 and circMTND5 backsplicing activity in LF-sperm tail, thus negatively affecting cirCNOT6L, circLZIC, and circL3MBTL4 biogenesis. To confirm this hypothesis, we carried out a RIP assay in CTRL and LF-spermatozoa, using FUS antibody (Fig. 2F). Relative to the use of IgG control, the results showed a significant (*P* < 0.01) fold enrichment of cirCNOT6L (13.45 f.c.), circLZIC (11.23 f.c.), and circL3MBTL4 (14.59 f.c.) when the anti-FUS antibody was used in CTRL-spermatozoa (Fig. 2F). Similarly, a significant (*P* < 0.01) fold enrichment of cirCNOT6L (4.3 f.c.), circLZIC (5.32 f.c.), and circL3MBTL4 (6.32 f.c.), lower than that observed in CTRL-spermatozoa, occurred in LF-spermatozoa (Fig. 2F). Conversely, a significant (*P* < 0.01) fold enrichment of circRASA3 (5.6 f.c.), circEIF2C2 (6.7 f.c.) and circMTND5 (4.3 f.c.) was observed in RIP experiments carried out in CTRL-spermatozoa SPZ (Fig. 2F). Nevertheless, a significant (*P* < 0.01) fold enrichment of circRASA3 (13.6 f.c.), circEIF2C2 (12.6 f.c.) and circMTND5 (13.5 f.c.), interestingly higher than that observed in CTRL-spermatozoa, occurred in LF-spermatozoa (Fig. 2F), confirming that FUS translocation in the sperm tail of LF-spermatozoa enhanced the endogenous backsplicing of sperm low-quality circRNAs.

### Pb, a representative heavy metal, induces the pathozoospermic phenotype onset in high-quality spermatozoa

On the basis of previous findings (Castellanos *et al.*, 2013; He *et al.*, 2016), we hypothesized that in LF-normozoospermic men seminal plasma, high levels of Pb may induce morpho-functional defects and circRNA landscape anomalies in high-quality spermatozoa. With this in mind, we carried out *in vitro* Pb experiments in high-quality spermatozoa collected from CTRL normozoospermic men. In detail, sperm cells were incubated *in vitro* with vehicle (Ctrl experimental group) or Pb (Pb experimental group) and used for morphological and molecular investigations.

**Figure 3.**
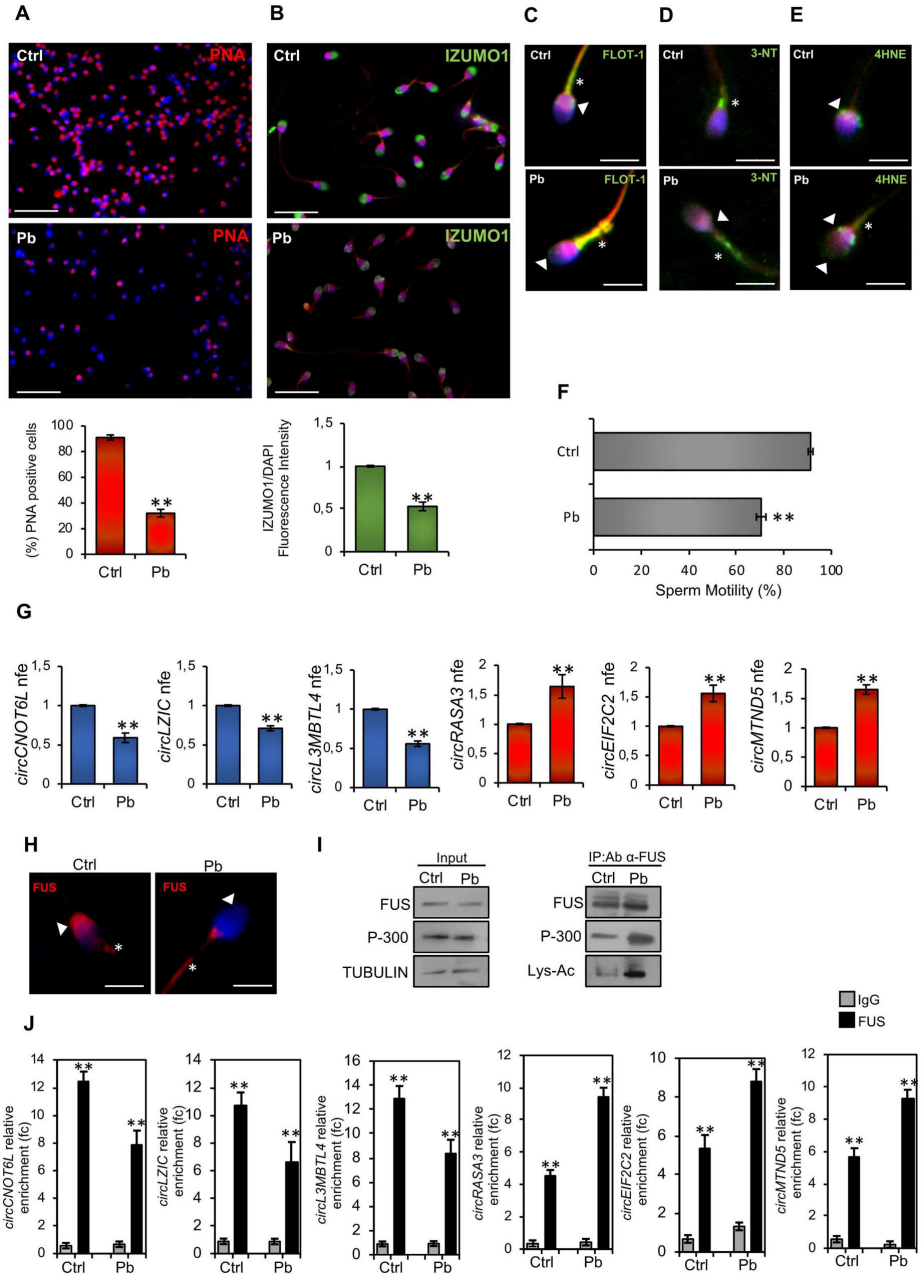
**A heavy metal drives the pathozoospermic phenotype in high-quality spermatozoa from normozoospermic men (men not living in the Land of Fires).** (**A**) Immunofluorescence analysis of PNA (red) (n = 6 different samples for each experimental group) and cellular counting of PNA-positive sperm cells in Ctrl- and Pb-spermatozoa (n = 10 different samples in triplicate for each experimental group). Nuclei were labelled with DAPI (blue). Scale bar: 50 μm. Data are expressed as percentage of positive cells on total and reported as mean ± SEM; **P < 0.01. (**B**) Immunofluorescence analysis of IZUMO1 (FITC-green) (n = 6 different samples for each experimental group) and immunofluorescence signal intensity analysis (n = 6 different samples for each experimental group) in Ctrl- and Pb-spermatozoa. Values are expressed as mean ± SEM; **P < 0.01. Immunofluorescence analyses of Flotillin-1 (FITC-green) (**C**), 3-NT (FITC-green) (**D**), and 4HNE (**E**) in Ctrl- and Pb-spermatozoa (n = 6 different samples for each experimental group). White arrowheads represent sperm head and neck localizations; white asterisks represent localizations in sperm midpiece. Nuclei were labelled with DAPI (blue); scale bar: 4 μm. (**F**) Motility assay in Ctrl- and Pb-spermatozoa (n = 10 different samples in triplicate for each experimental group). Data are expressed as the percentage of motile/live spermatozoa and reported as mean ± SEM; **P < 0.01. (**G**) Expression analysis of circRNAs (cirCNOT6L; circLZIC; circL3MBTL4; circRASA3; circEIF2C2; circMTND5) in Ctrl- and Pb-spermatozoa (n = 6 different samples in triplicate for each experimental group). qRT-PCR data were normalized using GAPDH, expressed as fold expression (nfe) and reported as mean value ± S.E.M. **P < 0.01. (**H**) Immunofluorescence analysis of FUS (red) in Ctrl- and Pb-spermatozoa (n = 6 different samples for each experimental group). Nuclei were labelled with DAPI (blue); white arrowheads represent FUS localization in sperm head; white asterisks represent FUS localization in sperm tail. Scale bar: 4 μm. (**I**) Western blot analysis of IP in Ctrl- and Pb-spermatozoa (n = 6 different samples for each experimental group) using FUS antibody. FUS-IP was analysed in comparison with Input protein extracts. (**J**) The enrichment levels of circRNAs (cirCNOT6L; circLZIC; circL3MBTL4; circRASA3; circEIF2C2; circMTND5) in the products of RIP (FUS-IP compared with IgG-IP) in Ctrl- and Pb-spermatozoa detected by qRT-PCR. Data are reported as mean ± SEM (n = 6 different samples for each experimental group in triplicate); **P < 0.01. Ctrl: control experimental group, spermatozoa incubated *in vitro* with vehicle; FUS: fused in sarcoma; 4HNE: 4-hydroxy-2-nonenal; IP: immunoprecipitation; 3-NT: 3-nitrotyrosine; PNA: peanut agglutinin; qRT-PCR: quantitative RT-PCR; RIP: RNA-binding protein immunoprecipitation. Applied statistical test: Student's *t*-test.

PNA staining was performed in Ctrl- and Pb-spermatozoa by immunofluorescence analysis to investigate Pb-dependent effects on acrosome morphology. As reported (Fig. 3A), Pb-spermatozoa showed a drastic reduction in the frequency of physiological acrosomal PNA signal. Accordingly, a significant reduction (*P* < 0.01) in the percentage of sperm cells with positive PNA signal occurred in Pb-spermatozoa (Fig. 3A), confirming that Pb treatment *in vitro* induced AR onset in high-quality spermatozoa. Similarly, IZUMO1 immunofluorescence analysis showed a widespread and lower IZUMO1 signal in Pb- than Ctrl-spermatozoa, as confirmed by fluorescence intensity analysis that highlighted a significant (*P* < 0.01) downregulation of IZUMO1 signal in Pb- when compared with Ctrl-spermatozoa (Fig. 3B).

Then, immunofluorescence analyses of Flotillin-1, 3-NT, and 4HNE were carried out in Ctrl- and Pb-spermatozoa in order to investigate potential Pb-dependent effects on plasma membrane fluidity and sperm oxidative state. In accordance with anomalous localizations in LF-spermatozoa, *in vitro* Pb treatment induced a high increase in Flotillin-1 signal in the midpiece region (Fig. 3C). In addition, NT and 4HNE signals appeared highly diffused throughout the midpiece region in Pb- when compared with Ctrl-spermatozoa (Fig. 3D and E), collectively demonstrating that *in vitro* Pb treatment negatively affected the lipid arrangement of the sperm plasma membrane and the oxidative state, potentially impacting sperm motility. To confirm this hypothesis, we performed a motility assay in Ctrl- and Pb-spermatozoa (Fig. 3F). In comparison with Ctrl group, a significant reduction (*P* < 0.01) in the percentage of motile spermatozoa was observed following Pb treatment *in vitro*, demonstrating that Pb directly modulates plasma membrane fluidity and ROS production, thereby affecting sperm motility.

To confirm if the pathological exposure to Pb contamination was responsible for the sperm circRNA impairment characterized in LF-spermatozoa, we analysed the expression levels of circRNAs related to high- (cirCNOT6L; circLZIC; circL3MBTL4) and low- (circRASA3; circEIF2C2; circMTND5) sperm quality in Ctrl- and Pb-spermatozoa by qRT-PCR analysis. Expression levels of all circRNAs related to sperm high quality were significantly lower (*P* < 0.01) in Pb- than Ctrl-spermatozoa (Fig. 3G). As expected, an inverse expression profile of circRNAs related to sperm low-quality was observed in Pb-spermatozoa; in fact, a significant increase (*P* < 0.01) in the expression of all circRNAs analysed occurred following *in vitro* Pb treatment (Fig. 3G). To verify the involvement of FUS protein in regulating this peculiar circRNA expression pattern, and in particular focusing on a potential responsiveness of FUS subcellular localization to Pb *in vitro* treatment, we carried out FUS immunofluorescence analysis in Ctrl- and Pb-spermatozoa. Consistent with our hypothesis, FUS localization was completely impaired following *in vitro* Pb treatment, as a clear translocation from sperm head to tail was observed in Pb-spermatozoa (Fig. 3H). Accordingly, IP experiments carried out in Ctrl and Pb-spermatozoa, separately, using FUS antibody, showed an increase of FUS/p-300 interaction rate in Pb-spermatozoa, not dependent on FUS and p-300 protein content, as confirmed by western blot analysis of input samples (total lysates isolated before the IP) (Fig. 3I). Interestingly, a significant increase in FUS acetylation was observed following Pb *in vitro* treatment, thus demonstrating that Pb contamination promoted FUS hyperacetylation *via* p-300 physical interaction and, in turn, its nucleo-cytosolic shuttling which enabled a compromised sperm circRNA profile. To experimentally validate our idea, we lastly carried out a RIP assay in Ctrl and Pb-spermatozoa, using FUS antibody (Fig. 3J). As reported, relative to IgG control, a significant (*P* < 0.01) fold enrichment of cirCNOT6L (12.46 f.c.), circLZIC (10.74 f.c.) and circL3MBTL4 (12.84 f.c.) was observed when the anti-FUS antibody was used in Ctrl-spermatozoa (Fig. 3J). Accordingly, a similar significant (*P* < 0.01) fold enrichment of cirCNOT6L (7.89 f.c.), circLZIC (6.66 f.c.) and circL3MBTL4 (8.3 f.c.) occurred in Pb-spermatozoa, although lower than that observed in Ctrl. Following Pb *in vitro* treatment, RIP experiments showed a significant (*P* < 0.01) fold enrichment of circRASA3 (4.51 f.c.), circEIF2C2 (5.34 f.c.) and circMTND5 (5.71 f.c.) in Ctrl-spermatozoa whereas a significant (*P* < 0.01) fold enrichment increase of circRASA3 (9.41 f.c.), circEIF2C2 (8,84 f.c.) and circMTND5 (9.32 f.c.) occurred in Pb- when compared with Ctrl-spermatozoa.

## Discussion

In recent decades, the Land of Fires has suffered an intense disposal of toxic waste, with a devastating effect on human reproductive health (Fattore *et al.*, 2006, 2008; Bergamo *et al.*, 2016; Esposito *et al.*, 2017; Lettieri *et al.*, 2020a,b; Alberti 2022): hence the urgency to identify bio-molecular markers for sperm quality, and circRNAs are very promising candidate molecules (Chioccarelli *et al.*, 2019; Ragusa *et al.*, 2019; Manfrevola *et al.*, 2020). The correlation between male infertility and environmental pollution has been widely investigated (Jeng, 2014; Easley *et al.*, 2015; Governini *et al.*, 2015; Gao *et al.*, 2017; Zhang *et al.*, 2018; Lombó *et al.*, 2019); however, a functional study aimed to define an impact on sperm circRNAs has not yet been performed.

The success of fertilization is ensured by the best performance of high-quality spermatozoa. Nevertheless, the morpho-functional and epigenetic characterization carried out here has unmasked the poor quality of these cells when collected from normozoospermic men living in the Land of Fires geographical area. As previously demonstrated (Bergamo *et al.*, 2016), the seminal plasma of these subjects also showed a considerable bioaccumulation of several toxic elements, heavy metals included.

In order to better understand if such a bioaccumulation may perturb sperm quality, we carried out several morphological analyses. LF-spermatozoa showed structural defects, such as tapered heads and bent tails, typically related to the teratozoospermia condition (Auger *et al.*, 2016). In addition, acrosome integrity, evaluated through PNA signal intensity and localization, was also assessed. A large number of LF-spermatozoa lost PNA signal or reduced it to a residue localized in the post-acrosomal region, suggesting a potential early AR onset. In accordance with this consideration, a significant reduction in the percentage of IZUMO1 positive cells was demonstrated in the LF group, as already revealed in human spermatozoa in the case of the AR (Gómez-Torres *et al.*, 2023). The pathozoospermic condition here hypothesized could be dictated by abnormal seminal steroid levels, as previously demonstrated (Chen *et al.*, 2019; Calogero *et al.*, 2000; Osadchuk *et al.*, 2023). In line with these data, we carried out a hormonal analysis in LF seminal plasma, observing increased levels of E_2_ and PG that well matched with previous findings.

Plasma membrane integrity of LF-spermatozoa was, then, ascertained through Filipin III and Flotillin-1 signals. Interestingly, an abnormal cholesterol accumulation as well as an anomalous lipid arrangement in lipid rafts were both observed, suggesting a strong impact on plasma membrane fluidity and on sperm motility, as a consequence. In this regard, oxidative stress has been reported to be implicated in the harmful effects on sperm motility and, furthermore, a direct correlation between toxicant exposure and sperm motility decline has been demonstrated (Naha and Manna 2007; Patra *et al.*, 2011; Almeida Lopes *et al.*, 2016; Kurkowska *et al.*, 2020). The increase of 3-NT and 4HN4 oxidative markers, in association with the motility reduction of LF-spermatozoa, clearly suggest the trigger of oxidative stress as a consequence of toxic element bioaccumulation in men living in Land of Fires.

An aspect still to be explored is how much damage ECs cause on the sperm epigenetic landscape, especially on circRNA cargo. This topic appears of great interest considering that sperm circRNAs are constantly acquiring key roles in the setting of sperm morpho-epigenetic high-quality (Chioccarelli *et al.*, 2019; Ragusa *et al.*, 2019; Manfrevola *et al.*, 2020, 2023).

Therefore, we decided to focus our attention on the Pb contaminant, in light of its predominant presence in industrial and urban waste, illegally released in Land of Fires. Our results demonstrated a direct correlation between seminal Pb levels and the decline of circRNAs related to high-quality spermatozoa, as well as an increase of circRNAs related to low-quality spermatozoa, strongly supporting our hypothesis that Pb exposure may promote this changed pattern. However, we cannot exclude a possible interplay among Pb and other heavy metals underlying this modulation. Further investigations are necessary to fully understand this issue. Nevertheless, the most exciting results, here reported for the first time, consist of sperm circRNA deregulation selectively correlated to their subcellular localization. In fact, all circRNAs related to high- and low-quality spermatozoa, previously reported to be differentially enriched in the sperm head and tail, respectively (Chioccarelli *et al.*, 2019), appeared also down- and up-regulated in LF-spermatozoa. At this point, we asked how ECs may preferentially promote a backsplicing mechanism of circRNAs related to low-quality spermatozoa, specifically localized in sperm tail. To answer this question, we focused our attention on FUS protein, the main RBP protein involved in the endogenous sperm backsplicing activity and primarily localized in sperm head (Chioccarelli *et al.*, 2021a). Considering that in amyotrophic lateral sclerosis neurodegenerative disease the acetylation of FUS protein at specific lysine residues, catalysed by p300 acetyl-transferase, results in cytoplasm mislocalization able to favour the pathological formation of stress granule-like inclusions (Arenas *et al.*, 2020), we hypothesized that such a molecular mechanism could occur in LF-spermatozoa as well. Accordingly, in these cells, a predominant FUS localization in sperm tail, associated with a tighter physical interaction with p-300 and an enhanced FUS acetylation, was observed and perfectly matched with our hypothesis. In addition, RIP experiments definitively demonstrated the increased FUS-dependent sperm low-quality circRNA biogenesis reinforcing, and thus confirming, that the exposure to toxic contaminants has promoted a poor circRNA landscape drawing in LF-spermatozoa. In other pathological conditions, Pb exposure has been associated with increased histone acetylation levels leading to apoptosis in cardiac tissues (Xu *et al.*, 2015). Intriguingly, p-300 protein is one of the main acetyl-transferases modulating histone acetylation in sperm cells during spermiogenesis, it has been found in mature spermatids, and it also participates in lysine residue acetylation of non-histone proteins (Pradeepa *et al.*, 2009; Arenas *et al.*, 2020; Chioccarelli *et al.*, 2020b). Furthermore, we showed expression of p-300 in human spermatozoa that appeared in agreement with previous findings in normozoospermic men (Kim *et al.*, 2014). Overall, these findings strongly suggested the hypothesis that Pb exposure was able to enhance p-300 activity towards its protein targets, including FUS. Similar molecular mechanisms may be driven by other heavy metals; this still remains an unexplored scenario.

To better appreciate how ECs may alter sperm physiology, and limiting this study to Pb bioaccumulation side effects, we set up an *in vitro* Pb-exposure experiment on high-quality spermatozoa collected from normozoospermic men, not living in Land of Fires, for the purpose of demonstrating that Pb exposure was able to induce the same acrosome anomalies, oxidative stress increase and sperm motility reduction observed in LF-spermatozoa. Such a similar result was also obtained in circRNA dynamics, molecularly driven by FUS subcellular localization. Indeed, Pb *in vitro* treatment exerted a drastic effect on nucleo-cytosol shuttling of FUS protein dependent on the interaction with p-300, and downstream on its increased acetylation state. Once again, FUS-RIP experiments gave us the final support of our hypotheses. Thus, Pb-induced FUS shuttling, disadvantaging the biogenesis of circRNAs related to high-quality spermatozoa and localised to the head subcellular compartment, promoted the biogenesis of a low-quality circRNA counterpart in the sperm tail.

Collectively, the results discussed here open the doors to a worrying new pathozoospermic condition, that is EC-dependent, in young men classified as normozoospermic. Land of Fires has been used as an optimal case study to assess this topic, with the results showing that high-quality spermatozoa assumed several morphological traits as in teratozoospermia and an enhanced biogenesis of low-quality circRNAs, molecularly driven by head-to-tail FUS shuttling, as a consequence of toxicant exposure.

## Data Availability

The datasets in this study are available from the corresponding author upon reasonable request.
